# RETRACTED: Block et al. Fluorinated Analogs of Organosulfur Compounds from Garlic (*Allium sativum*): Synthesis, Chemistry and Anti-Angiogenesis and Antithrombotic Studies. *Molecules* 2017, *22*, 2081

**DOI:** 10.3390/molecules30092005

**Published:** 2025-04-30

**Authors:** Eric Block, Benjamin Bechand, Sivaji Gundala, Abith Vattekkatte, Kai Wang, Shaymaa S. Mousa, Kavitha Godugu, Murat Yalcin, Shaker A. Mousa

**Affiliations:** 1 Department of Chemistry, University at Albany, State University of New York, Albany, NY 12222, USA; 2 The Pharmaceutical Research Institute, Albany College of Pharmacy and Health Sciences, Rensselaer, NY 12144, USA; 3 Department of Physiology, Veterinary Medicine Faculty, Uludag University, Bursa 16059, Turkey

The Journal retracts the article “Fluorinated Analogs of Organosulfur Compounds from Garlic (*Allium sativum*): Synthesis, Chemistry and Anti-Angiogenesis and Antithrombotic Studies” [1], cited above.

Following the publication of a correction [2] to this article [1], further concerns were brought to the attention of the Editorial Office by Albany College of Pharmacy and Health Sciences relating to the validity of the sub-images presented in Figures 5 and 6 in the “Biological Studies” portion of the article.

Adhering to our standard procedure, an investigation was conducted by the Editorial Office and Editorial Board, and, supported by information provided by the Albany College of Pharmacy and Health, the Editorial Board agreed with the institutional assessment that the replacement images provided in the above-mentioned correction could not be appropriately validated due to the lack of supporting raw material and thus could not be considered as satisfactorily resolving the identified concern. As a result, the Editorial Board has lost confidence in the reliability of the biological studies’ findings and decided to retract this publication [1]. This retraction supersedes the previously published correction [2] and is carried out as per MDPI’s retraction policy (https://www.mdpi.com/ethics#_bookmark30). Some of the authors (Eric Block, Benjamin Bechand, Sivaji Gundala, Abith Vattekkatte, and Kai Wang) have indicated their intention to submit part of this study as a new submission titled “Fluorinated Analogs of Organosulfur Compounds from Garlic (*Allium sativum*): Synthesis and Chemistry” to *Molecules*.

This retraction was approved by the Editor-in-Chief of the journal *Molecules*.

Eric Block agrees to this retraction. The remaining authors did not provide a comment on this decision.

## References

[1] Block E., Bechand B., Gundala S., Vattekkatte A., Wang K., Mousa S.S., Godugu K., Yalcin M., Mousa S.A. (2017). RETRACTED: Fluorinated Analogs of Organosulfur Compounds from Garlic (*Allium sativum*): Synthesis, Chemistry and Anti-Angiogenesis and Antithrombotic Studies. Molecules.

[2] Block E., Bechand B., Gundala S., Vattekkatte A., Wang K., Mousa S.S., Godugu K., Yalcin M., Mousa S.A. (2023). Correction: Block et al. Fluorinated Analogs of Organosulfur Compounds from Garlic (*Allium sativum*): Synthesis, Chemistry and Anti-Angiogenesis and Antithrombotic Studies. *Molecules* 2017, *22*, 2081. Molecules.

